# A phase 2 quasi-experimental trial evaluating the feasibility, acceptability, and potential effectiveness of complex nursing intervention focused on QoL assessment on advanced cancer patients with palliative care needs: study protocol

**DOI:** 10.1186/s40814-017-0196-x

**Published:** 2017-11-13

**Authors:** Gianluca Catania, Annamaria Bagnasco, Alessio Signori, Paola Pilastri, Marta Bottino, Claudia Cervetti, Milko Zanini, Giuseppe Aleo, Loredana Sasso

**Affiliations:** 1 Department of Health Sciences, School of Medical and Pharmaceutical Sciences, University of Genova, Via Pastore 1, 16132 Genoa, Italy; 2 Department of Medical Oncology, IRCCS AOU San Martino—IST Istituto Nazionale per la Ricerca sul Cancro, Largo R. Benzi 10, 16132 Genoa, Italy; 3 Hospice Unit, Gigi Ghirotti not-for-profit association, Corso Europa 50, 16132 Genoa, Italy

**Keywords:** Quality of life, Complex intervention, Palliative care, Patient-reported outcome measure, Cancer patient, Outcome measurement, Outcome assessment, Development, Implementation, Nurse-led intervention, Nursing-sensitive patient outcomes, Patient-centered outcome measure

## Abstract

**Background:**

Palliative care (PC) is an approach that improves the quality of life (QoL) of patients and their families facing the problem associated with incurable terminal disease. A number of QoL assessment tools have been validated in PC and their use described for research purposes, to support clinical practice, and as part of the quality improvement programs. There is a paucity of evidence on the implementation of a nursing intervention focused on QoL assessment in PC practice.

The aim of this study is to model and determine the feasibility of a nursing complex intervention focused on QoL assessment in PC practice.

**Methods:**

The intervention will be evaluated through a quasi-experimental non-equivalent comparison group before-after study design. This project can be classified as phases 1–2, according to the Medical Research Council (MRC) framework for the development and evaluation of complex interventions.

The study setting will take place in two inpatient hospice units in Italy. The study sample will be constituted of 39 multidisciplinary healthcare professionals and a sample of 46 advanced cancer patients admitted to hospices during the implementation of the intervention.

**Discussion:**

This study will generate information to address the implementation of QoL measurement in palliative care practice. Findings of this study will be used to inform a phase 3 trial according to the MRC framework.

**Trial registration:**

ISRCTN41201864 retrospectively registered.

## Background

Although quality of life (QoL) assessment is a central concept in palliative care (PC) practice [1], the standardized application of clinical interventions focused on assessing QoL in clinical practice is limited. In addition, current research is insufficient to determine how to implement such interventions and determine their impact on patient outcomes [2, 3].

QoL has been described as the gap between individual’s expectations and their perception of a given situation [4], and its management entails the use of a QoL tool to screen and then monitor QoL changes over time. Recently, authors of a systematic review on facilitators and barriers to the successful implementation of Patient-Reported Outcome Measures (PROMs) in PC recommended tailoring this implementation according to the setting, having a coordinator throughout the implementation process, offering all staff an educational program, and recognizing the ongoing cognitive and emotional processes in each individual [5].

During the previous decade, a number of QoL measurement intervention components targeted at staff and practice in PC have been suggested such as choosing a tool, identifying a leader of the project, and staff training [6]. In addition, a European-funded research project—the PRISMA-FP7 project—introduced standards for the measurement of outcomes in PC [7], and more recently, an evidence-based clinical guidance on how to respond to patient-reported outcomes measurement in clinical care was proposed [8]. These initiatives indirectly emphasized both the inherent complexity of such interventions and the need for clinicians to respond to the patients’ complex needs. International scientific associations contribute to a growing body of guidelines claiming that QoL dimensions need to be comprehensively assessed and documented using available standardized scales [9].

In 2010, a systematic review conducted to determine the availability of PROMs to assess QoL in PC identified many measurement instruments and concluded that there was no agreement on how quality of life should be measured [10]. A subsequent systematic review evaluated to what extent interventions focused on QoL assessment improve PC patient outcomes revealed that overall implementation interventions focused on QoL assessment in PC practice do result in improved patient outcomes. Although included studies suffered from small sample size and selection, attrition, and performance bias, findings showed that such interventions can have a moderate practical significance on symptoms, psychosocial dimensions, and overall QoL [2].

Therefore, we designed an evidence-based intervention focused on QoL measurement in PC practice developed according to the Medical Research Council (MRC) framework for developing and evaluating complex interventions [11]. We are interested in understanding the feasibility of implementing the intervention in PC practice to inform the design of a phase 3 randomized controlled trial.

### Aims

The aim of the present study is to model and determine the feasibility of a nursing complex intervention focused on QoL assessment in PC practice and developed according to the MRC framework for developing, implementing, and evaluating complex interventions [11].

## Methods/design

**Fig. 1:**
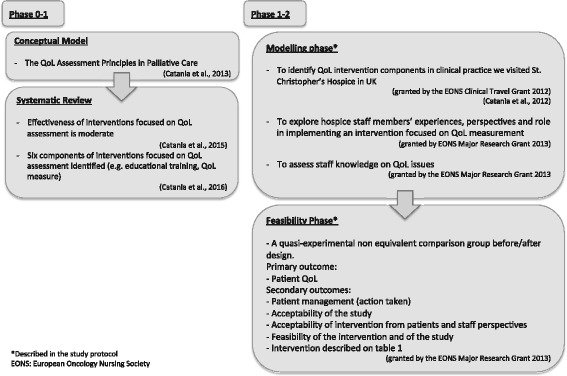
Flowchart of the INFO-QoL study

Our study is designed to improve PC patients’ QoL (i.e., prevent/manage impaired QoL) during admission to an inpatient hospice unit. Flowchart of the entire intervention focused on QoL measurement (INFO-QoL) project and this study protocol (phases 1–2) are shown in Fig. 1 **.**

### Operational definitions

For the purpose of this study, QoL was defined as the gap between a person’s life experience and expectations [4]. Addressing the problem (i.e., impaired QoL) begins by assessing patient’s QoL, and the information collected will serve as a basis to formulate realistic goals [7].

Our primary hypothesis is that patients, who will be cared for by hospice staff that have implemented a nurse-led complex intervention focused on QoL measurement, will have a significantly better QoL compared with patients cared for by staff that provide traditional care.

According to the MRC framework, the primary objective of the study is to model the INFO-QoL intervention (phase 1) and determine the feasibility, acceptability, and potential effectiveness of the INFO-QoL intervention (phase 2).

The secondary objective is to pilot methods for a phase 3 randomized controlled trial including sample size, the power of the study, recruitment, follow-up, suitability of measures, and documentations.

The research questions are the following:

1. Are the procedures of the INFO-QoL conveniently performed as planned to yield information on patients’ needs within a reasonable time frame?
2. Is the INFO-QoL acceptable to hospice staff and their patients?
3. What is the potential effectiveness of the implementation of the INFO-QoL in terms of patients’ QoL and QoL-related management actions taken by PC staff per patient?

### Conceptual model

The study will be based on the conceptual model of *The QoL Assessment Principles in Palliative Care* developed according to phase 0 of the MRC framework. It consists of four sections, for a total of 11 principles to be considered in developing and/or evaluating clinical interventions focused on QoL assessment in PC. The model has been proposed as a methodological and ethical standard to be considered when developing and evaluating clinical interventions focused on QoL assessment in PC. Section one includes three principles related to “the problem” (i.e., a QoL impairment), section two includes two principles dealing with the assessment tool, section three includes one principle focusing on the treatment/intervention, and section four includes five principles dealing with “the clinical intervention focused on QoL assessment” [12] (Table 1).

**Table 1:** The Qol assessment principles in palliative care

The problem (the Qol impairment)
1. The problem should be a serious condition for the patient either in terms of prevalence (e.g., pain, depression) and/or distress for the patient (e.g., itch, hiccup) or the result of late detection and management of the problem (e.g., a new or unusual distressing symptom occurred over the disease trajectory).
2. The problem should be highly unlikely to be reported by the patient or recognized by the professional if not actively assessed.
3. The trajectory of the problem should be sufficiently understood to assure a timely assessment to anticipate and appropriately address the problem.
The assessment tool
4. A validated, reliable, and sensitive-to-change tool for detecting and measuring the problem should be available.
5. The tool should be practical and easy to use, and questions must not be distressing for the patients.
The treatment-intervention
6. There should be an appropriate treatment/intervention for patients with the recognized problem.
The clinical intervention focused on Qol assessment
7. There should be an agreed policy on which a problem (or a problem with a certain degree of impairment) has to be addressed with appropriate treatment or intervention.
8. It should be possible for the tool to be appropriately administered by professionals trained in the procedure.
9. The treatment-intervention for patients with QoL impairments should be available, with appropriately trained professionals.
10. The cost of problem-finding (including all the steps from the administration of the tool until the treatment-intervention has been delivered in full) should be economically justified.
11. QoL assessment should be a continuing process rather than a one-off assessment.

Adapted from Catania et al. [12]

### Intervention design (theoretical phase)

The development of the INFO-QoL was carried out through several stages hereafter described (1) We conceptualized that any intervention focused on QoL assessment is a complex intervention and as such includes many components that form a coherent structure that links the intervention to patient’s QoL [12]. (2) We developed the conceptual model of *The QoL Assessment Principles in Palliative Care* by adapting the WHO Screening Principles framework [13] to QoL assessment in PC [12]. (3) We identified the components and the extent of the outcomes resulting from the interventions focused on QoL measurement by systematically reviewing the literature [2, 14]. (4) We identified the key elements that led to successful QoL measurement and then determined whether such components could be used in Italy by undertaking a study visit at St. Christopher’s Hospice in UK granted by the European Oncology Nursing Society (EONS) through the Clinical Travel Grant 2012. After asking the experts in the hospice, they emphasized that components such as staff education, measurement timeframe, designated person in charge of the process, and results discussed during unit staff briefing were the key elements they addressed before starting up QoL measurement [15]. Such components need to be locally adapted before their implementation in Italy. The final version of the INFO-QoL is described in Table 2.

**Table 2:** The intervention focused on quality of life measurement

1. Name of intervention
The INtervention FOcused on Quality of Life Measurement (The INFO-QoL)
*2.* Goals of intervention
a. Ultimate goal: to prevent/manage impaired quality of life in advanced cancer patients with palliative care needs in hospice setting and promote measurement of patient’s quality of life b. Immediate goals: i. To reduce prevalence and/or severity of patient’ s problems/needs ii. To identify patients at risk of developing impairments in QoL dimensions iii. To increase staff awareness of problems/needs, their trajectory, and appropriate intervention to address them iv. To inform individual care plans based on impaired QoL dimensions and local policies
*3.* Components and activities
a. Component 1: ensure that treatment plans and evaluations focus on patient rather than disease i. Goal: to educate staff about QoL issues and about interventions that promote a better QoL ii. Activities: discuss the following topics: – What is QoL in palliative care patients? – What are the dimensions that contribute to a person’s QoL? – What are the determinants of changes in self-assessment of QoL? – How staff should propose, use, and score QoL tools to patients – How palliative care staff can effect changes in patients to improve their QoL b. Component 2: the Palliative Outcome Scale (POS) as an integral component of the intervention i. Goal: patient and family education on using outcome measures ii. Activities: present the purpose of QoL assessment to patients and their families; educate patients on using the POS c. Component 3: QoL measurement using the POS i. Goal: to promote cohesive, coordinated patient- and family-centered care ii. Activities: screening (i.e., baseline) and monitoring individual patients (morning shift), record individual and overall scores on individual INFO-QoL form, and discuss results to inform clinical decision during unit staff briefing
4. Mode of delivery
a. Component 1: small group education session (6–8 palliative care nurses) i. Use combination of written and verbal presentation and group discussion ii. Written presentation: case-based materials, self-study binders, poster iii. Verbal presentation: evidence on QOL issue in palliative care iv. Group discussion: elicit case discussions to focus on issues related to patients’ QoL b. Component 2: nurses-patient and family face-to-face interaction i. Use combination of written material and verbal communication ii. Written materials, such as handout on QoL issues, and poster with large print and simple language hanging in the inpatient hospice unit iii. Verbal communication using a slow rate of speech, simple and common words, short sentences, and a teach-back technique c. Component 3: individual QoL measurement i. Nurse team leader is responsible to manage the measurement (i.e., schedule the measurement, prepare documentation, assign patient to nurse or nursing assistant, oversee completion of QoL measurement) ii. Nurse or nursing assistant who perform the QoL measurement motivates patient to improve compliance iii. Staff discusses results and gives feedback of results and care plan to patient
5. Dose
a. Component 1: self-study binders given 2 weeks before the education bundle program session; one small group education session of 3-h duration (repeated at different time points to allow all staff to participate) b. Component 2: education session of about 10/15-min duration c. Component 3: measurement within day 3 from admittance, days 8–10, day 15 and then once a week; patient feedback at the same day of the measurement

### Study population

The study will be conducted on a convenience sample of two hospice units of the urban district of Genoa in the north of Italy: the Gigi Ghirotti Bolzaneto Hospice is a 12-bed nonprofit hospice (Gigi Ghirotti Hospice—Association for Research and Treatment of Pain and Palliative Care), and the Maria Chighine Hospice is a 12-bed public hospice (IRCCS AOU San Martino—IST—Teaching Hospital and Cancer Research Centre). In both of these hospice units, healthcare professionals were educated and trained in PC and communication in end-of-life care and are using the Liverpool Care Pathway [16]. According to the definition of the European Association for Palliative Care, “they admit patients in their last phase of life when treatment in the hospital is not necessary and care at home or in nursing home is not possible” [17]. The National Health System covers all or part of the costs of the care for the public and nonprofit hospice, respectively. The hospice staff of the Ghirotti Hospice includes 1 physician, 10 nurses, and 6 nursing assistants, and the Maria Chighine Hospice includes 3 physicians, 12 nurses, and 7 nursing assistants.

The sample will consist of all the PC team members within the two hospices during the modeling and feasibility phase. During the 6-month period of the feasibility phase (pre-test = 3 months; post-test = 3 months), all adult cancer patients newly admitted to the hospice units will be invited to participate in the study and they will be included after giving their informed consent. Patients too ill to receive the intervention or unable to give informed consent either due to cognitive impairment or because they are unable to understand Italian will be excluded.

### The modeling phase

The modeling phase will be conducted through a qualitative approach:

1. Semi-structured face-to-face interviews to explore hospice nursing staff members’ experiences and their role in implementing a nurse-led intervention focused on QoL measurement
2. Focus groups with nursing staff members to explore their perspective about a draft version of the INFO-QoL received a week before the scheduled focus group sessions
3. A questionnaire survey to assess staff knowledge on QoL in PC. The questionnaire was developed according to relevant QoL literature. An academic working group of experts in PC was set up with the aim of developing the questionnaire, because a measure to gather information from PC staff about their knowledge on QoL issues in PC area did not exist. Four academic members from the University of Genoa (Italy) and the Trinity College of Dublin (UK) discussed and developed the questionnaire. The Nursing Role Effectiveness Model [18] was used to guide the development of the questionnaire. The model is based on the structure-process-outcome model of quality of care [19]. For item generation, the structure component comprehends nurses’ knowledge on quality of life in PC, the process component includes the role of nurses in PC and specifically QoL assessment-related activities, and finally, the outcome component was conceptualized as patient QoL. Sources used for item generation include a comprehensive review of the published literature, the quality of life [20], and the Outcome Measurement in Palliative Care—The Essential [7]. Guided by the conceptual framework, items were selected and assembled according to Dillman’s techniques for questionnaire construction [21, 22]. Content validity was systematically assessed and quantified according to the process described by Lynn [23]. Twenty-six international experts were invited to take part in a QoL expert panel, and 14 agreed to participate. Each of them received an email to explain the objective of the project, a structured procedure for the evaluation of content validity, and a link to the online evaluation of the draft version of the questionnaire consisting of 25 items. Each expert was invited to rate independently the relevance of each item using a four-point Likert scale (1: not relevant; 2: somewhat relevant; 3: quite relevant; 4: very relevant). The Content Validity Index (CVI) was computed for each item on a scale (I-CVI), as well as for the overall scale (S-CVI). For each item, the I-CVI was computed as number of experts giving a rating of either 3 or 4, divided by the total number of experts. The items that had I-CVIs of 0.78 or higher were retained [24]. Of the 25 items, eight were deleted and three were revised and included in the questionnaire. The final version of the INFO-QoL questionnaire was made up of 17 items with a S-CVI/Ave of 0.81 computed using the averaging method [24].

The results of the questionnaire survey will be used to inform the QoL education bundle as a component of the intervention (Component 1 of the INFO-QoL—Table 2).

As a result of this phase, the intervention will be modeled and linked to a new local procedure.

### The exploratory trial (assessing feasibility and piloting method)

As the INFO-QoL is being implemented throughout the unit and it will be difficult to deliver the intervention randomly to some patients but not to others, the feasibility stage will have a quasi-experimental non-equivalent comparison group before/after design. Although randomized controlled trials represent the gold standard, and quasi-experimental designs have a weakened confidence in making causal assertions that the results occurred because of the intervention, quasi-experimental designs can also provide useful information to establish whether (and to what extent) an intervention is effective. A non-equivalent comparison group design is the strongest of the quasi-experimental designs and can provide good evidence for nursing practice. Quasi-experimental designs are more feasible and more commonly conducted for nursing studies in clinical settings. This design will enable the researcher to pilot the components and methods and the feasibility of the INFO-QoL, in order to assess its acceptability in practice and determine whether the INFO-QoL is delivered according to the study protocol. It will also offer the opportunity to determine potential effectiveness of the INFO-QoL in improving patient QoL.

To partially address selection bias, the INFO-QoL described in Table 2 will be introduced randomly into unit 1 (intervention unit), while patients and staff in unit 2 (comparison unit) will continue with traditional clinical practice. By gathering pre-test data, we can compare the equivalence of the two groups on antecedent variables before introducing of the independent variable [25]. The study will inform methods for the future phase 3 trial in determining sample size and the power of the trial, recruitment, follow-up, and suitability of measures. For the purpose of this study, the type and quality of care delivered by sites will not be assessed: it will be assumed to be at least comparable between the two hospice units.

### Randomization

The unit of randomization is the hospice unit. To prevent performance bias before randomly allocating the experimental intervention in one of the two units, the collection of pre-test data will occur within a three-month period in both units before making any change. Subsequently, to reduce the selection bias, the INFO-QoL will be implemented randomly in “unit 1” (intervention unit), while patients and staff in the “unit 2” (comparison unit) will continue with the standard clinical practice.

Randomization will be performed independently by using a computer-generated sequence. The randomization procedure will be centralized and managed by an independent statistician at the coordinating center of the study. All study investigators, personnel, and participants will be unaware of the randomization procedure.

### Sample size

According to Cocks and Torgerson [26], this pilot trial needs a sufficient sample size, so that if our observed difference between the two groups, in the pilot trial, is zero, then the upper confidence limit will exclude the estimate that is considered “clinically significant” in the planned definitive trial. We will consider a small effect size of 0.3 and choose a confidence of 80% (that will satisfy the need for reasonable certainty for trial decision-making but would be small enough to ensure a study within a reasonable budget and timeframe). A sample size of 17 patients for unit/period produces a one-sided 80% confidence interval with a distance from the difference in means to the one-sided limit that is equal to 0.200 when the estimated standard deviations are 1.00 and 1.00. A 30% attrition rate based on rates identified in a recent systematic review [27] will lead to the inclusion of 6 additional patients for a total of 23 patients unit/period.

## Outcomes

### The modeling phase (phase 1 study)

Both qualitative and quantitative data will be collected from both staff groups through semi-structured face-to-face interviews and focus groups. A set of questions to stimulate responses from the staff members will be generated according to Patton’s [28] recommendations and included in an interview protocol. The interviews will be used to explore nursing staff’s past experience and role in implementing an intervention focused on QoL measurement, whereas focus groups with nursing staff members will enable them to explore their perspective about a draft version of the INFO-QoL they will receive a week before the scheduled focus groups sessions. Two researchers with experience in palliative care will lead the interviews.

In both units, staff members’ knowledge on QoL will be assessed through a web survey using the INFO-QoL questionnaire before randomizing the site that will receive the intervention and after implementing the intervention.

Demographic information will be collected, including age, gender, level of education, professional role, years in their professional role, and years in PC.

### The exploratory trial (phase 2 study)

#### Feasibility of intervention

Feasibility will be measured in terms of time taken to organize and perform the intervention and to train professionals on QoL.

Also, fidelity of intervention will be assessed relating to deviations from the procedures and uncompleted measurements (with reasons), recorded using structured staff self-report checklists.

In addition, professionals will be asked to rate their competence and level of confidence in delivering the intervention at different points in time.

#### Feasibility of the study

Study feasibility will be assessed in terms of time taken to obtain patients’ informed consent and recruitment and patient dropout rates.

#### Acceptability of the study

Acceptability will be evaluated in terms of proportion of eligible patients (who were approached about the study) accepting to participate in this study.

##### Acceptability of intervention from patients’ perspective

Once the delivery of the intervention is completed, acceptability will be examined using a semi-structured questionnaire designed and developed for this study. Patients will be asked to appraise the intervention in terms of appropriateness and usefulness in identifying their needs using a QoL tool and to suggest ways to enhance its acceptability and to identify any missing elements [29].

##### Acceptability of intervention from staff’s perspective

Acceptability will be assessed using a semi-structured interview including five open questions.

After implementing the intervention—at the end of a 3-month period—staff members’ acceptability will be explored in terms of relevance, appropriateness, and usefulness in addressing QoL of palliative care patients.

To enhance the completeness and acceptability of the intervention, staff members will be asked to suggest ways to modify any aspects of the intervention and their professional views use the INFO-QoL as a standard practice in the unit.

Prior to staff interviews, the intervention (INFO-QoL) will be provided to staff in the intervention unit, giving a fundamental overview of QoL as the main focus of palliative care. Staff in the intervention unit will then be asked:

1. How would you comment on the relevance of the intervention in addressing palliative care patients’ QoL?
2. How would you appraise the intervention overall in terms of its appropriateness in addressing palliative care patients’ QoL?
3. How would you appraise the intervention overall in terms of its usefulness in addressing palliative care patients’ QoL?
4. Would you suggest modifying any components and/or activities of the intervention?
5. Would you recommend using the INFO-QoL as a standard practice within the unit?

#### Potential effectiveness

QoL will be assessed by collecting data before (period I: 3 months) and after (period II: 3 months) the intervention and comparing QoL scores in the intervention and comparison unit using the Italian version of the Palliative Outcome Scale (POS). The QoL assessment will be performed within day 3 (T0) from admittance, on day 8–10 (T1), and then on day 15 (T2). To reduce detection bias, a trained research assistant—who will be blind to intervention or comparison status of the unit—will administer the POS to patients in the comparison unit. Staff will receive full disclosure of the study purpose. This outcome measure has been originally validated in advanced cancer patients in the UK. Cronbach’s alpha for internal consistency was 0.65 and kappa for reliability ranged from 0.8 to 0.62. The POS includes ten items on physical, psychological, and spiritual dimensions. Responses to each of the ten questions range on a scale from 0 to 4. A score of 0 indicates “not a problem at all” and 4 indicates “overwhelming” for the patient. The completion time is 7 min [30]. The psychometric properties of the Italian version of the POS were acceptable, Cronbach’s alpha coefficient was 0.65, and intra-class correlation coefficient was 0.72 [31].

Demographic patient information will be collected, including age, gender, education level, marital status, primary cancer site, and length of time since diagnosis. We assume that if patients are admitted to hospice with a primary diagnosis of cancer, they will all have advanced stage cancer.

#### Patient management

Patient management will be evaluated using an adapted version of the composite patient management score by Detmar et al. [32]. All QoL-related management actions taken by staff for patients will be calculated by summarizing all the actions taken. Data will be collected from medical and nursing charts and ad hoc documentation, which will comprise the terminology included in the QoL tool (i.e., the POS) to collect actions delivered to patients for each of impaired QoL dimensions resulting from the POS score. Data on these actions will be elements of care pathways including but not limited to medications, interventions, nutrition and dietary, vital signs, diagnostic tests, referrals and consultations, patient and family counseling, and education.

## Ethics statement

The INFO-QoL research project was approved by the IRCCS AOU San Martino—IST Regional Ethics Committee (Study #335REG2014). All participants—both healthcare professionals and patients—will be asked to sign an informed consent form. Preliminarily, they will receive a one-page study information sheet, which will be discussed, and then they will be asked if they would be willing to participate in the study. Patients will also be informed that refusing to participate do not affect the care they need and that they could withdraw their consent at any time. Also, participants will be informed that their data will be protected from inappropriate disclosures according to Italian laws and regulations. Healthcare professionals invited to participate will be advised that declining does not lead to any penalty or loss of benefits otherwise entitled.

## Discussion

Despite numerous publications on the issue of patient QoL, there is a paucity of evidence on how best to address QoL assessment in clinical practice. The findings of our project could add important evidence-based knowledge on this topic. Therefore, our results can help build knowledge about what components to include in modeling an intervention focused on QoL assessment, which is useful for other implementation projects to learn from. A clinical intervention focused on QoL assessment provides innovation for oncology and palliative nursing in several ways: (1) fostering the simultaneous care model; (2) ensuring that the voices (needs) of people with advanced cancer are heard; (3) helping at screening and monitoring patients’ needs to fully personalize care through evidence-based nursing interventions; (4) supporting the evidence-based practice movement to ensure quality of service, effectiveness and accountability, and personalized care; (5) facilitating identification of what works and what does not work in delivering clinical nursing interventions aimed both at measuring and improving patients’ QoL; and (6) assuring that patients’ wishes and preferences will be recorded and understood by health professionals delivering care to them.

Early initiation of palliative care has been proved to improve QoL significantly in the outpatient setting [33]. Although this study will be focused on hospice inpatient units, it will potentially also contribute to gain an in-depth insight into developing and implementing complex interventions focused on QoL assessment in settings other than hospice units, such as hospital and community settings [29, 34]. It may contribute to improving patient’s QoL in advanced disease trajectory as well. A systematic literature review showed that QoL assessment leads to improved delivery of patients’ physical, psychological, and social needs [2] and that more rigorous studies are necessary to establish a routine clinical practice approach for identifying and addressing patients’ unmet needs. The innovative approach identified and piloted in our study may help to fulfill this need, as we have rigorously applied the MRC framework to palliative care patients in clinical practice, in order to address these key issues identified in the literature.

This study may also contribute towards the development of research in palliative care practice, addressing a relevant topic for patients, families, and healthcare professionals. The strength of the study is related to the multiple components of the intervention based on data currently available. The structured approach of the intervention may provide a method for identifying informed care plans and improving patients’ outcomes. Healthcare palliative professionals involved may find the intervention acceptable for delivery in clinical practice after receiving formal training in all the preliminary phases of the study. Moreover, as a consequence of the staff’s commitment, training/education received, and expectation of improved patient outcomes, they may decide to maintain use of the INFO-QoL intervention as standard practice beyond the duration of this study.

## Abbreviations

CVI: Content Validity Index
EONS: European Oncology Nursing Society
I-CVI: Item level Content Validity Index
INFO-QoL: INtervention FOcused on QoL measurement
MRC: Medical Research Council
PC: Palliative care
POS: Palliative Outcome Scale
PROMs: Patient-Reported Outcome Measures
QoL: Quality of Life
S-CVI: Scale level Content Validity Index
WHO: World Health Organization
